# Formulation and Development of Nanofiber-Based Ophthalmic Insert for the Treatment of Bacterial Conjunctivitis

**DOI:** 10.3390/ijms25179228

**Published:** 2024-08-25

**Authors:** Eszter Farkas, Houssam Abboud, Nándor Nagy, Bálint Hofmeister, Eszter Ostorházi, Bence Tóth, Balázs Pinke, László Mészáros, Romána Zelkó, Adrienn Kazsoki

**Affiliations:** 1 Center of Pharmacology and Drug Research & Development, University Pharmacy Department of Pharmacy Administration, Semmelweis University, Hőgyes Endre Street 7-9, H-1092 Budapest, Hungary; farkas.eszter5@gmail.com (E.F.); houssam.abboud@phd.semmelweis.hu (H.A.); zelko.romana@semmelweis.hu (R.Z.); 2 Department of Anatomy, Histology and Embryology Semmelweis University, Tűzoltó Street 58, H-1094 Budapest, Hungary; nagy.nandor@semmelweis.hu; 3 Department of Medical Microbiology, Semmelweis University, Nagyvárad Square 4, H-1089 Budapest, Hungaryostorhazi.eszter@semmelweis.hu (E.O.); 4 Department of Pharmaceutics, Semmelweis University, Hőgyes Endre Street 7, H-1092 Budapest, Hungary; toth.bence@semmelweis.hu; 5 Department of Polymer Engineering, Faculty of Mechanical Engineering, Budapest University of Technology and Economics, Műegyetem Rkp. 3, H-1111 Budapest, Hungary; pinke@pt.bme.hu (B.P.); meszaros@pt.bme.hu (L.M.)

**Keywords:** electrospinning, nanofiber, ophthalmic insert for bacterial eye infection, solid-state characterization, in vitro dissolution test, antibacterial study, cytotoxicity test

## Abstract

A novel ophthalmic delivery system utilizing levofloxacin-loaded, preservative-free, nanofiber-based inserts was investigated. Polyvinyl alcohol (PVA) and Poloxamer 407 (Polox)were employed as matrix materials, while hydroxypropyl-beta-cyclodextrin (HP-β-CD) was a solubilizer. The formulations were prepared via electrospinning and characterized for fiber morphology, drug dissolution, cytotoxicity, and antimicrobial activity. Scanning electron microscopy confirmed uniform fibrous structures. Fourier Transform Infrared spectroscopy and X-ray diffraction analyses demonstrated the amorphous state of levofloxacin within the fibers. In vitro dissolution studies revealed a rapid (within 2 min) and complete drug release, with higher HP-β-CD levels slightly delaying the release. Cytotoxicity tests showed increased HP-β-CD concentrations induced irritation, that was mitigated by sodium hyaluronate. The antimicrobial efficacy of the nanofibers was comparable to conventional eye drops, with lower minimum inhibitory concentrations for most tested strains. The nanofibrous formulation prepared from a PVA–Polox-based viscous solution of the drug:CD 1:1 mol ratio, containing 0.4% (*w*/*w*) sodium hyaluronate) was identified as a particularly promising alternative formulation due to its rapid and complete dissolution, good biocompatibility, and effective antimicrobial properties. Its gelling properties indicate that the residence time on the eye surface can be increased, potentially reducing discomfort and enhancing therapeutic outcomes. The nanofibrous formulations enhanced antimicrobial efficacy, providing a preservative-free alternative that minimizes the potential eye irritation that might occur because of the preservative agent and reduces the administrated dose frequency by extending the drug’s retention time on the eye’s surface. Subsequently, it improves patients’ adherence, which would reflect positively on the bioavailability. The levofloxacin-HP-β-CD nanofibers demonstrate promise as an alternative to traditional eye drops, offering advantages in solubility, stability, and patient compliance for ocular infection treatment.

## 1. Introduction

Fluoroquinolone-based ocular antibacterial formulations are considered primary choices for treating ocular bacterial infections due to the high sensitivity of isolates, particularly *Staphylococcus* and *Streptococcus*, to fluoroquinolones [1,2]. Their mechanism is to inhibit the DNA gyrase and topoisomerase IV. This antibacterial group is divided into various generations, including ciprofloxacin (2nd generation), levofloxacin (LEVO) (3rd generation), and moxifloxacin (4th generation). The 4th generation fluoroquinolones, like moxifloxacin and gatifloxacin, exhibit an enhanced antibacterial efficacy but may also induce more significant toxicity to corneal epithelial cells than other fluoroquinolones [3]. LEVO, a hydrophobic 3rd generation fluoroquinolone with broad-spectrum antibacterial activity against gram-positive and gram-negative bacteria, demonstrates the most negligible cytotoxicity among these newer generations [4].

LEVO is an efficient and widely used medication for common eye infections such as blepharitis, conjunctivitis, and keratitis due to its effective antibacterial properties and low toxicity compared to other fluoroquinolones. The medication is available in various ocular dosage forms, including eye drops and eye gel or eye ointment. The recommended dosage regimen for 0.5% LEVO eye drops is one drop every 1–2 h for the first three days, followed by one drop every 4–5 h [5]. The recommended dosage is four times daily for 0.3% LEVO levofloxacin eye gel [6].

Despite the advantages of conventional dosage forms of LEVO, such as eye drops and eye gel or ointment, there are still some drawbacks. The treatment efficacy highly depends on the patient’s adherence to achieve the required drug concentration in the applied tissue. The high dosage frequency required in these administration regimens can negatively affect patient compliance and drug efficacy [7,8]. Additionally, other factors such as the complex anatomy of the eyeball, fast drainage of the drug from the eye surface due to lacrimation, nasolacrimal drainage, blinking, and other barriers presented by the eye can further affect the drug’s bioavailability. These factors can make it challenging to achieve the desired therapeutic outcomes with conventional dosage forms [9].

Due to the mentioned challenges, the bioavailability of LEVO in ocular applications is notably low, primarily due to inadequate drug permeation across ocular membranes and the brief duration the drug remains at the application site. In optimal conditions, only about 5% of the administered dose reaches the target tissues [9,10]. Furthermore, being a hydrophobic drug, LEVO faces difficulties in formulation as eye drops, leading to inferior ocular bioavailability compared to hydrophilic drugs [11]. Despite these limitations, significant efforts and research have been dedicated in recent years to address these issues by developing novel ocular drug delivery systems utilizing various techniques. These advancements aim to improve the bioavailability of ocular medications, including inserts and implants. Although the availability of such systems in the market is limited, the field continues to attract considerable attention from researchers and the scientific community, focusing on the anterior segment and the posterior segment of the eye [12,13,14,15,16].

Various drug delivery systems have been created and employed to enhance the delivery of medications. These include utilizing a film composed of hyaluronic acid and polyvinyl alcohol (PVA) to transport dexamethasone and LEVO to the cornea and through the sclera, potentially targeting the posterior eye segment while ensuring the controlled release of both lipophilic and hydrophilic drugs [17]. Additionally, biodegradable poly (lactic-co-glycolic acid) nanoparticles have encapsulated LEVO for sustained ocular drug delivery [18]. Other methods involve delivering LEVO through topical chitosan/β-glycerophosphate-based hydrogel [19], chitosan nanoparticles in an in situ gel system to enhance corneal residence time and improve antibacterial efficacy [20], and sustained-release bioadhesive minitablets for once-daily ocular administration [21]. Furthermore, approaches like chitosan-coated poly(lactic-co-glycolic acid) nanoparticles [22], chitosan nanoparticles based on sulfobutyl-ether-beta-cyclodextrin SBE-β-CD loaded with LEVO [23], a pH-triggered in situ gel for sustained delivery using hydroxypropyl methylcellulose) and sodium alginate [24], and a thermosensitive glycol chitosan-based hydrogel as a topical ocular drug delivery system have been developed to improve drug delivery efficiency and effectiveness [25].

Ocular inserts have garnered significant interest among researchers due to their versatility in being crafted from a diverse range of polymers, which can effectively improve drugs’ bioavailability by prolonging drug residence time and regulating release patterns. As solid formulations, they offer enhanced drug stability compared to liquid forms. Additionally, these formulations eliminate the necessity for preservative agents, reducing the risk of irritation associated with such additives [26]. An important technique for manufacturing ocular inserts is the electrospinning method, known for producing sub-micrometer fibers [27]. This method allows for the encapsulation of various substances, including hydrophilic and hydrophobic chemical drugs, proteins, antibodies, nucleic acids, and small, interfering ribonucleic acids [28,29,30]. These substances can be encapsulated together or separately in distinct compartments using different polymers such as PVA, a synthetic polymer known for its excellent mechanical strength and biodegradability [31]. 

Furthermore, the characteristics of the produced fibers can be altered using various techniques, such as enhancing the solubility of hydrophobic drugs and improving the adhesion of the fibers. This can be accomplished by employing poloxamers, a group of neutral synthetic polyoxyethylene polypropylene block copolymers with hydrophilic ends and hydrophobic cores. Poloxamers facilitate the encapsulation of hydrophobic drugs within an aqueous framework [32]. Additionally, solubility can be increased by incorporating cyclodextrins (CD), which possess a structure comprising a hydrophobic core and a hydrophilic shell [33]. For instance, hydroxypropyl-β-cyclodextrin (HP-β-CD), a commonly used CD in ocular formulations, is known for its eye-friendly properties and enhancing the solubility of drugs [34,35].

**Figure 1:**
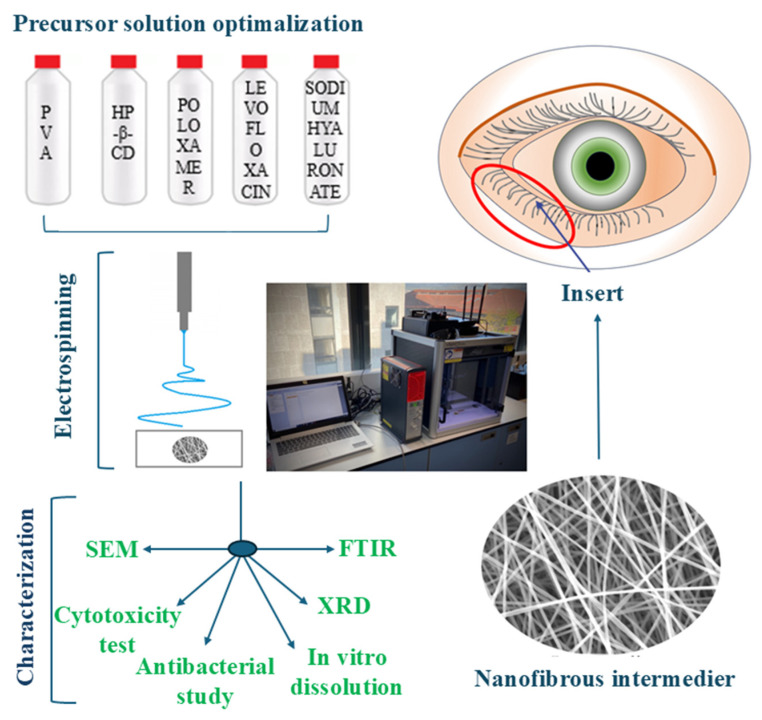
Schematic representation of the project objectives.

The main objective of the present study was to develop LEVO-loaded, soluble, preservative-free, nanofiber-based ophthalmic inserts, using PVA as the base of the medical device, poloxamer 407 (Polox) to enhance the mucoadhesive property of the system and to formulate in situ gel, and HP-β-CD as a solubilizer and stabilizer. Another goal was to examine how the excipients influence the electrospinning process, the characteristics of the fibers, the drug dissolution, and the cytotoxicity and antibacterial properties of the obtained nanofibrous inserts (Figure 1).

## 2. Results

### 2.1. Morphological Analysis of the Samples

**Figure 2:**
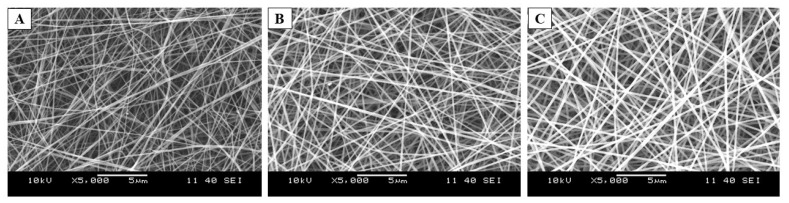
Scanning electron microscopy (SEM) images of the polyvinyl alcohol (PVA): poloxamer 407 (Polox) (8:2 mass ratio) based, electrospun samples of total polymer concentrations 10% (*w*/*w*) (**A**), 12% (*w*/*w*) (**B**), and 14% (*w*/*w*) (**C**), respectively (Magnification: 5000×).

The preformulation study started with the total polymer concentration optimization of PVA:Polox8:2 (m:m). The ratio of these two polymers was kept constant and was determined based on our previous study. The polymer concentration was examined in three levels: 10-, 12-, and 14% (*w*/*w*) polymeric solution was used for the electrospinning process. The morphology of the resulting samples could be seen in the SEM images (Figure 2). After the optimization of the process parameters, fibrous samples could be obtained. Only in the case of the examined lowest concentration could some bead-like formations be observed (Figure 2A). As the active ingredient’s water solubility needed to be enhanced by the addition of HP-β-CD (which will enhance the viscosity of the precursor solutions) for the further drug-loaded fiber formation, the medium total polymer concentration was selected. 

The suitability of precursor solutions containing LEVO (3% (*w*/*w*)) for electrospinning processes with varying LEVO: HP-β-CD molar ratios (1:1 and 1:1.5) and two concentrations of sodium hyaluronate (0.2% and 0.4% *w*/*w*) was investigated. Table 1 summarizes the applied flow rate, voltage, and effective distance (the distance between the collector and the end of the needle) of the different composition precursor solutions used for the fiber formation process. 

**Figure 3:**
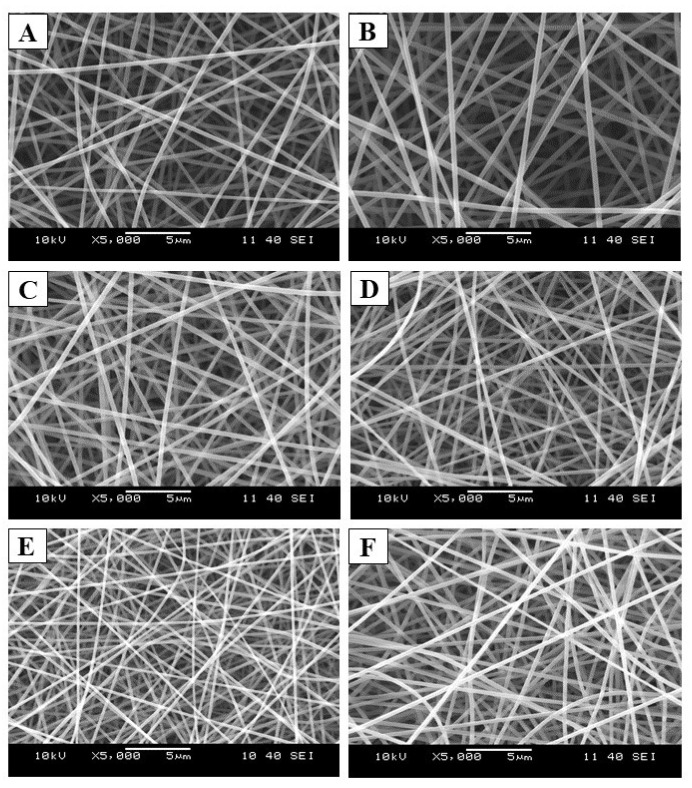
Scanning electron microscopic (SEM) images of the electrospun samples that used F4 (**A**), F5 (**B**), F6 (**C**), F7 (**D**), F8 (**E**), and F9 (**F**) precursor solutions for the fiber formation process (Magnification: 5000×).

By optimizing the electrospinning process parameters, well-defined, round-shaped samples were produced on the collector. As illustrated in Figure 3, a clear fibrous structure without any beads or film-like elements was observed in all samples. The SEM analysis confirmed the absence of visible heterogeneity in both samples.

Table 2 summarizes the average fiber diameters with standard deviations (SD) and the brief morphological evaluation of the electrospun samples. The results clearly show that in the case of the neat fiber (F1, F2, and F3), the fiber diameter increased with the increasing total polymer concentration, while a remarkable difference or tendency was not observed in the case of the drug-loaded samples. 

### 2.2. Solid-State Characterization of the Fibrous Samples

Fourier Transform Infrared (FTIR) spectroscopy and X-ray diffraction (XRD) measurements were conducted to characterize the polymer-based macromolecular systems.

**Figure 4:**
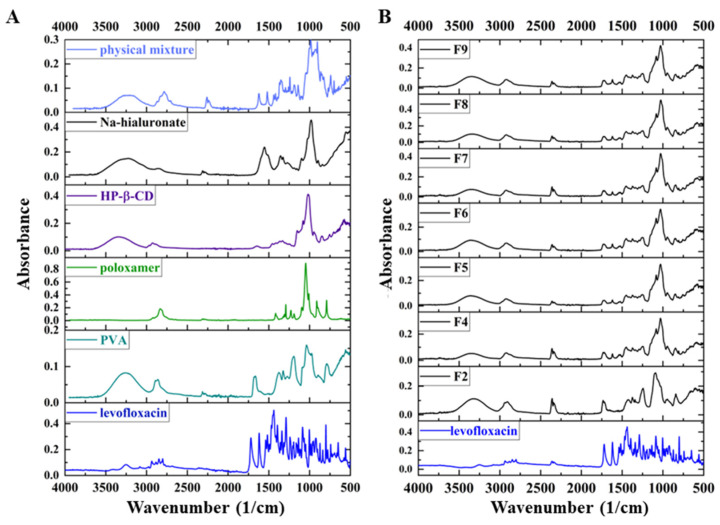
FTIR spectra of the components of the nanofibers and the physical mixture (**A**) and the prepared different compositions of nanofibrous samples and the levofloxacin (LEVO) (**B**) between 4000–500 cm^−1^.

FTIR spectroscopy was used to elucidate the molecular structure and functional groups in LEVO and other excipients. The FTIR spectrum of LEVO exhibited several characteristic absorption bands corresponding to specific functional groups, offering valuable insights into its chemical properties (Figure 4). The most prominent feature in the FTIR spectrum was observed at 1731 cm^−1^, attributed to the carbonyl (C=O) stretching vibration. Significant absorption bands in the region of 1500–1600 cm^−1^ were ascribed to the C=C stretching vibrations of the aromatic rings within the LEVO molecule, confirming the presence of aromatic systems essential for the biological activity of fluoroquinolones. Additionally, N-H stretching vibrations were detected in the 3100–3500 cm^−1^ range, crucial for identifying the amine functional groups, which are integral to the drug’s interaction with bacterial targets. These amine groups are consistent with fluoroquinolones’ known mechanisms of action, which involve interference with bacterial DNA synthesis. Furthermore, C-H stretching vibrations were observed around 2800–3000 cm^−1^, indicating the presence of aliphatic and aromatic hydrogen atoms. These vibrations contribute to the overall stability and solubility of the compound in various solvents, influencing its pharmacokinetic properties. Additional spectral features included skeletal vibrations of the piperazine ring, typically observed in the region of 1000–1300 cm^−1^, and C-O stretching vibrations associated with alcohol or ether functionalities, appearing around 1000–1200 cm^−1^.

In the spectra of the physical mixture, the most intensive characteristic peaks of LEVO can be observed, while in the spectra of the fibrous structure, a lack of sharp peaks related to the active pharmaceutical ingredients can be noted (Figure 3B). Unlike crystalline materials, where limited rotational–vibrational transitions are allowed, amorphous materials permit several transitions. The absorbed energy is dissipated among numerous transitions, leading to the broadening and merging of the spectrum because of the crystalline–amorphous transition.

**Figure 5:**
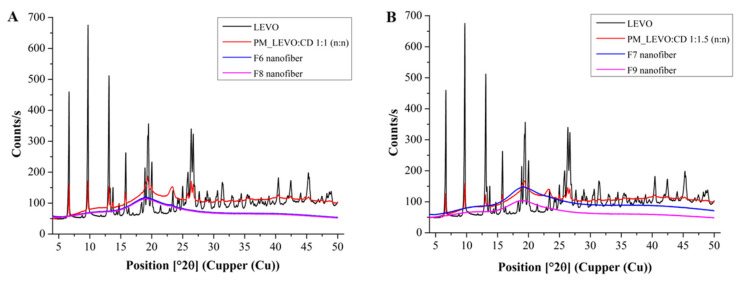
Power X-ray patterns of levofloxacin (LEVO), physical mixture, and drug-loaded nanofibers of LEVO–hydroxypropyl-beta-cyclodextrin (LEVO:CD) 1:1 (**A**) and 1:1.5 (n:n) (**B**), respectively.

XRD also analyzed the samples to confirm the formation of amorphous solid dispersion, which is a more sensitive method. In the X-ray diffractograms of physical mixtures, the characteristic peaks of the LEVO are observable, along with the broad peaks of the polymers and CD (Figure 5). In the physical mixture of LEVO: CD 1:1 (n:n), the characteristic peaks of the LEVO are more intensive since the LEVO concentration on the powder is higher, but also observable in the spectra of higher CD concentration (Figure 5B). Only diffuse peaks with the lack of any high-intensity peaks belonging to LEVO characterize the XRD patterns of the fibrous samples independently of the applied LEVO: CD mol ratio and the concentration of sodium hyaluronate.

The FTIR and the more sensitive XRD measurement results also suggested that amorphous solid dispersions were formed because of fiber formation, regardless of composition.

### 2.3. In Vitro Dissolution Study 

**Figure 6:**
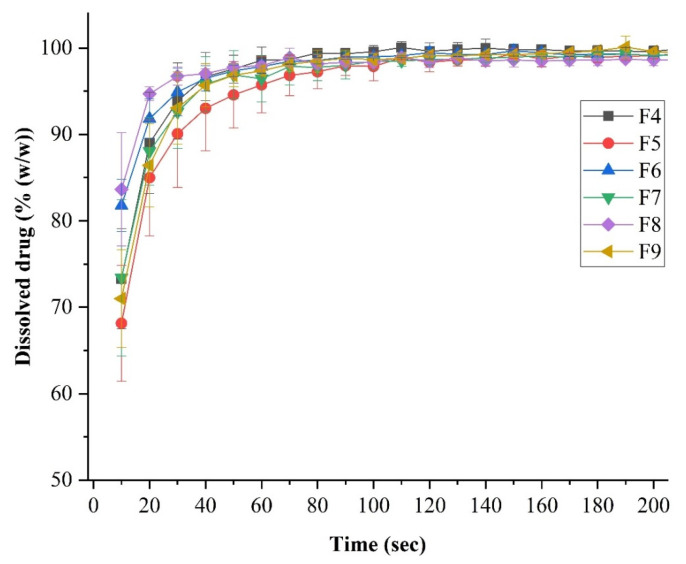
In vitro dissolution analysis of levofloxacin (LEVO)-loaded fibrous samples was carried out at a phosphate buffer of pH = 7.4, where the curves depict the average and deviation of the three parallel measurements.

Figure 6 summarizes the results of the dissolution test of LEVO-containing fibrous samples. The in vitro LEVO release was rapid and complete from each formulation. Within 100 s, all the active ingredient was dissolved. Higher HP-β-CD levels resulted in a slightly longer drug release. However, it can be said that remarkable differences could not be observed, and in the case of each formulation, a fast drug release was achieved. No clear trend was observed in the variation of Na-hyaluronate concentration, likely due to the minimal amount of this excipient present in the formulation.

### 2.4. In Vivo Cytocompatibility Test

**Figure 7:**
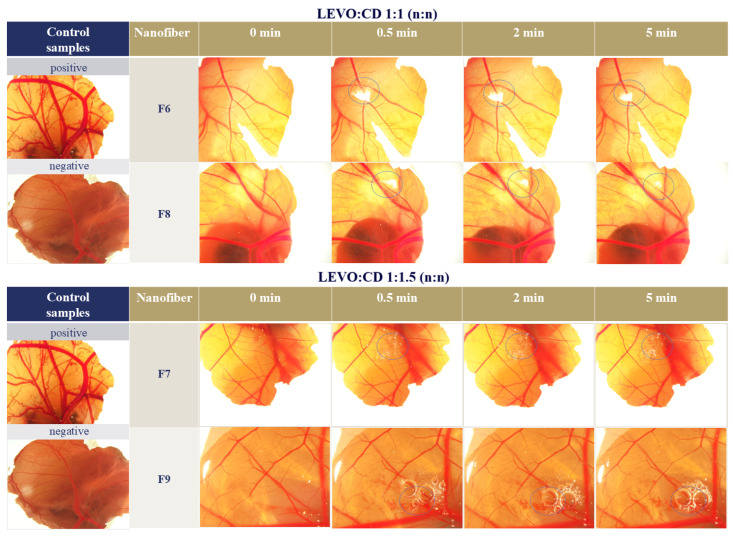
Images of the chorioallantois membrane (CAM) after placing different levofloxacin (LEVO)-loaded nanofibers of levofloxacin–hydroxypropyl-beta-cyclodextrin (LEVO:CD) 1:1 (F6 and F8) and 1:1.5 (n:n) (F7 and F9) and the negative and positive control (phosphate buffer salina (pH = 7.4) and 2M NaOH, respectively). The sodium hyaluronate concentration was 0.2% (*w*/*w*) for F6 and F7, and 0.4% (*w*/*w*) for F8 and F9 precursor solutions. The blue dashed circle indicates the position of the nanofibrous sample placed on the CAM.

In the case of the F6 and F8 formulations, no irritation and coagulation could be observed. With the increasing CD concentration (F7 and F9), irritation could be observed, whereas when the amount of sodium hyaluronate was increased, the irritation appeared later (Figure 7).

Quantitative analyses were performed using the scoring system by analyzing the extent of irritation based on specific indicators, typically hyperaemia, hemorrhage, and coagulation [36]. Table 3 summarizes the cumulative score and assessments of the irritation potential of different LEVO-loaded nanofibrous samples.

### 2.5. Results of the Aseptic Sample Preparation

**Figure 8:**
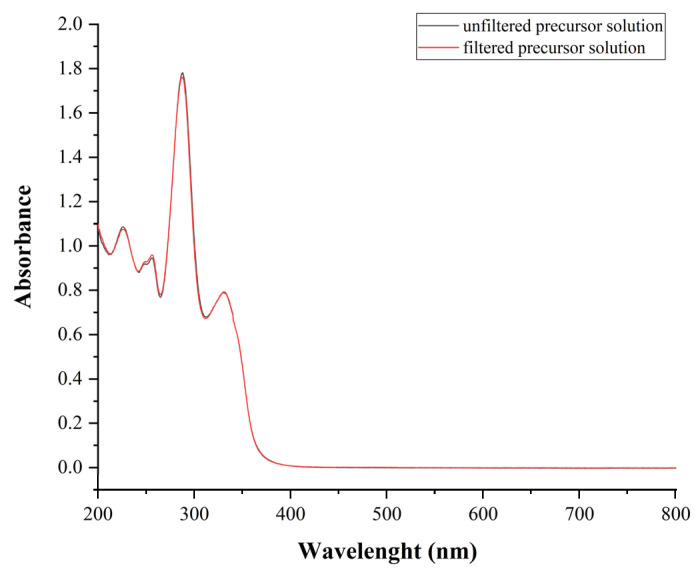
UV-Vis spectra of the unfiltered (black line) and filtered (red line) precursor solution between 200–800 nm.

The UV spectra of the unfiltered and filtered polymeric precursor solution revealed that the active materials could not be adsorbed to the membrane (Figure 8). Furthermore, no microorganism contamination was detected under the study’s circumstances. The results showed that the method was suitable for aseptic sample preparation.

### 2.6. Antimicrobial Study of the Fibrous Samples

#### 2.6.1. Disc Diffusion Method 

The antimicrobial efficacy of the LEVO, LEVO:CD aqueous solutions of 1:1 and 1:1.5 mol ratios, and the nanofibrous formulations (F6, F7, F8, F9) was evaluated using the disc diffusion method against five bacterial strains: *Streptococcus pneumoniae* (*S. pneumoniae*), *Moraxella catarrhalis* (*M. catarrhalis*), *Haemophilus parainfluenzae* (*H. parainfluenzae*), and two strains of *Neisseria gonorrhoeae* (*N. gonorrhoeae*). The zones of inhibition were measured in millimeters for each formulation, and Table 4 summarizes the results.

The results indicate that incorporating CD into the LEVO-loaded nanofibers generally enhances or maintains antimicrobial activity across different bacterial strains. The slight improvements that were observed in certain formulations, such as LEVO 1:1 against *S. pneumoniae* and *N. gonorrhoeae 1*, suggest that CD may improve the solubility and release kinetics of LEVO. For *M. catarrhalis*, the higher concentration formulations (particularly F7) demonstrated greater effectiveness than LEVO alone. This discrepancy highlights the need for further optimization of the nanofiber’s composition. In the case of *H. parainfluenzae* and *N. gonorrhoeae 2*, the results were consistent across all formulations and commercial discs, indicating the robust effectiveness of LEVO, regardless of the presence of CD.

These findings support the potential of LEVO-loaded nanofibers as a viable option for enhancing the efficacy of levofloxacin in antimicrobial applications.

#### 2.6.2. Determination of the MIC Values

The formulations’ Minimum Inhibitory Concentration (MIC) was determined. The MIC is the lowest concentration of an antimicrobial agent that inhibits the visible growth of a microorganism after incubation. It is crucial in microbiology and pharmacology to assess antimicrobial agents’ efficacy against specific microorganisms (*Escherichia coli* (*E. coli)*, *Staphylococcus aureus* (*S. aureus*), *Pseudomonas aeruginosa* (*P. aeruginosa*)). Table 5 summarizes the results of the MIC values.

In the case of *E. coli* bacteria, the MIC values for LEVO complexes and nanofibrous samples were significantly lower (<0.125 µg/mL) compared to LEVO alone (<0.25 µg/mL), indicating enhanced efficacy of the CD complexes and nanofibrous formulations.

The MIC of S. aureus for LEVO was between 8 and 16 µg/mL. LEVO complexes showed significantly reduced MIC values. F8 (0.25 µg/mL) exhibited the lowest MIC, indicating superior efficacy, followed by F7 and 9 (0.5 µg/mL) and F6 (16 µg/mL).

In the case of P. aeruginosa, the MIC for LEVO was 4 µg/mL. The LEVO complexes and nanofibrous formulations maintained this MIC value, apart from LEVO 1:1.5, which had a slightly higher MIC value.

The disc diffusion and MIC results collectively demonstrate the enhanced antimicrobial activity of LEVO when complexed with cyclodextrin and incorporated into nanofibrous formulations. The CD complexes and nanofibrous samples generally exhibited larger inhibition zones and lower MIC values than LEVO alone, suggesting improved drug delivery and efficacy.

The formation of an inclusion complex between the LEVO and CD enhances the solubility and stability of LEVO, resulting in increased antimicrobial activity, as evidenced by the reduced MIC values and larger inhibition zones.

The nanofibrous samples, particularly those with higher sodium hyaluronate content (F7 and F9), demonstrated superior efficacy against most bacterial strains. The increased surface area and sustained release properties of nanofibers likely contributed to their improved performance.

Among the formulations, F7 exhibited the highest efficacy in the disc diffusion tests, while Formulation 8 showed the lowest MIC values for *S. aureus,* indicating its potential as the most effective formulation.

#### 2.6.3. Determination of Minimum Bactericidal Concentration (MBC)

The MBC results provide insight into the bactericidal activity of the various LEVO formulations. The MBC values indicate the lowest concentration of the antimicrobial agent required to kill 99.9% of the bacterial population. The MBC data for different formulations are summarized in Table 6. 

For *E. coli*, the MBC for LEVO was 4 µg/mL, which was lower than the MBC values for the LEVO complexes and most nanofibrous samples (8 µg/mL). However, F7 showed an MBC equivalent to that of the LEVO solution (4 µg/mL), indicating that this particular nanofibrous formulation has a comparable bactericidal effect on *E. coli*. This suggests that while the cyclodextrin complexes and other nanofibrous formulations require higher concentrations to achieve the same bactericidal effect, F7 remains highly effective.

In the case of *S. aureus*, all LEVO solutions, including the cyclodextrin complexes and the eye drop formulation, demonstrated an MBC of 32 µg/mL. However, formulations F7, F8, and F9 exhibited higher MBC values (64 µg/mL), indicating that these nanofibrous samples are less effective at killing *S. aureus*. This could be attributed to factors such as drug release rate or drug-polymer interactions within the nanofiber matrix, which may affect the availability of the active drug at the site of infection.

For *P. aeruginosa*, the LEVO solution and all the LEVO:CD solutions as well as most of the nanofibrous formulations exhibited an MBC of 8 µg/mL. Notably, the eye drop formulation demonstrated a significantly lower MBC (1 µg/mL), indicating superior bactericidal activity against *P. aeruginosa* compared to the other formulations. This exceptional efficacy could be due to the enhanced solubility and bioavailability of LEVO in the eye drop formulation, facilitating more effective bacterial eradication.

The MBC results reveal distinct differences in bactericidal efficacy among the various formulations. While LEVO and Formulation 7 showed the lowest MBC for *E. coli*, suggesting high efficacy, the higher MBC values for *S. aureus* in formulations F7, F8, and F9 indicate a need for further optimization. The consistent MBC values for *P. aeruginosa* across most formulations, except for the significantly more effective eye drop, underscore the importance of formulation-specific factors in determining antimicrobial potency.

In conclusion, the MBC results suggest that while the nanofibrous formulations exhibit promising antimicrobial properties, their efficacy varies depending on the bacterial strain and specific formulation. The eye drop formulation demonstrated exceptional efficacy against *P. aeruginosa*, and the F7 nanofiber showed promising results against *E. coli*. These findings highlight the potential of these formulations for targeted antimicrobial therapy.

#### 2.6.4. Time–Kill Curve Measurements

**Figure 9:**
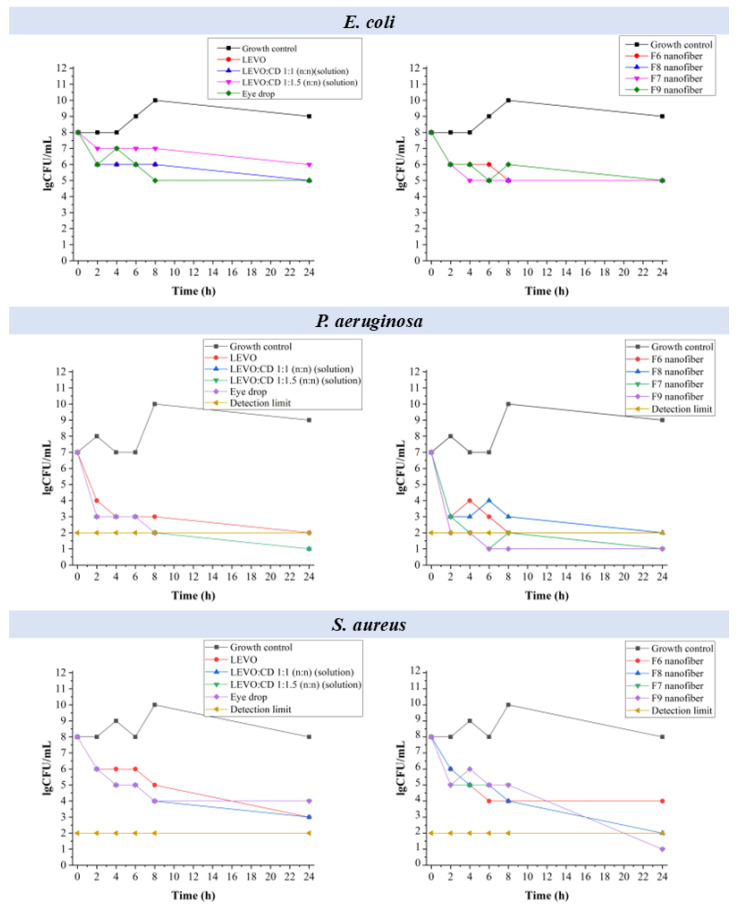
Time–kill plots demonstrating the effects of starting inoculum on the activities of different levofloxacin (LEVO)-containing formulations against *Escherichia coli* (*E. coli*), *Staphylococcus aureus* (*S. aureus*), and *Pseudomonas aeruginosa* (*P. aeruginosa*).

Time–kill curve measurements were performed to evaluate the bactericidal efficacy of the various LEVO formulations. This method involves monitoring the reduction in viable bacterial counts over 24 h, providing insight into the rate and extent of the bacterial killing by each formulation. These measurements aimed to compare the nanofibrous inserts’ antimicrobial performance with conventional eye drop formulations, aiming to identify a potential alternative treatment with improved bioavailability and patient compliance. The measured number of viable bacteria (colony-forming units, CFU) at various time points was summarized in Figure 9**.**

In the case of *E. coli*, the solutions showed a gradual decrease in bacterial count, falling below the detectable limits within 24 h. A rapid and sustained decrease in bacterial count was observed for the eye drop, maintaining low levels throughout 24 h.

In the case of the nanofibers (F6, F7, F8, and F9), a significant reduction in bacterial count was obtained, with F7 and F9 (higher sodium hyaluronate) performing slightly better, approaching the effectiveness of the eye drop formulation.

In the case of the LEVO solutions, rapid bactericidal activity was demonstrated for *P. aeruginosa*, achieving undetectable levels within 4 h. The eye drop was the most effective, maintaining undetectable levels within 4 h. The nanofibrous formulations (F6, F7, F8, and F9) had effective bactericidal activity, with all the formulations reaching undetectable levels by 24 h, showcasing potential as a viable alternative to eye drops.

For the solutions, a steady decrease in bacterial count and a significant reduction without reaching undetectable levels could be observed in the case of S. aureus. The eye drop was found highly effective, achieving undetectable levels within a few hours. The nanofibers (F6, F7, F8, and F9) showed a substantial reduction in bacterial count, with F7 and F9 showing slightly better performance, demonstrating the potential to match the eye drop’s efficacy.

So, it can be concluded that the eye drop formulation demonstrated the most potent and rapid bactericidal effect across all tested strains. However, the nanofibrous formulations, mainly F7 and F9 with higher sodium hyaluronate content, showed significant antimicrobial activity and promise as an alternative to eye drops. Given the issues of bioavailability and patient compliance associated with eye drops, the nanofibrous inserts offer a promising alternative, potentially providing sustained drug release and improved therapeutic outcomes. 

## 3. Discussion

The polymer concentration for electrospinning PVA nanofibers was successfully optimized, resulting in uniform fibrous structures with reduced bead formation as the polymer concentration increased. Structural uniformity is critical for consistent drug release, as non-uniformities can lead to variable drug loading and release rates. A morphological analysis confirmed that higher concentrations of polymer resulted in better-defined fibers, essential for the reliable performance of the delivery system.

One of the key points of this research was to enhance the solubility and stability of LEVO, a commonly used antibiotic. The incorporation of cyclodextrins (CDs), specifically HP-β-CD, significantly improved the solubility of LEVO. This improvement was reflected in the antimicrobial efficacy tests, where the LEVO formulations demonstrated superior antibacterial activity compared to LEVO alone. Their enhanced solubility likely facilitates better drug availability and penetration, crucial for effective treatment, especially of ocular infections.

Antimicrobial efficacy tests revealed that the nanofibrous formulations, particularly those with higher sodium hyaluronate content, exhibited strong bactericidal properties. Notably, one of the nanofibrous formulations showed antibacterial efficacy very similar to that of the eye drop formulation. This similarity is significant, as the nanofibrous insert offers distinct advantages over conventional eye drops. The application of the nanofibrous insert can enhance the residence time of the drug on the ocular surface, thereby increasing bioavailability. This prolonged residence time is particularly beneficial for sustained drug release, reducing the frequency of administration required and potentially improving therapeutic outcomes.

Additionally, the nanofibrous formulation is preservative-free, which reduces the risk of preservative-induced ocular irritation and toxicity, a common concern with the repeated use of eye drops. This feature, combined with the convenience of less frequent dosing, has the potential to improve patients’ adherence to the treatment regimen. Improved adherence is crucial for achieving the desired therapeutic efficacy, especially in chronic conditions requiring long-term treatment.

The cytotoxicity test, utilizing the hen’s egg test on the chorioallantois membrane (CAM) of 9-day-old chicken embryos, provided important insights into the safety profile of the formulations. It was observed that higher concentrations of HP-β-CD induced irritation, evidenced by hyperemia, hemorrhage, and coagulation on the CAM. This suggests that while HP-β-CD is effective in enhancing drug solubility, its concentration needs careful optimization to minimize cytotoxic effects. Interestingly, the addition of sodium hyaluronate was found to mitigate these adverse effects. Sodium hyaluronate is known for its biocompatibility and protective properties, likely contributing to the reduction in irritation by forming a more lubricating and protective layer on the ocular surface.

## 4. Materials and Methods

### 4.1. Materials

The LEVO was obtained from Merck Ltd. (Budapest, Hungary). The polyvinyl alcohol (PVA, Mowiol^®^ 18–88 with an average molecular weight Mw ~ 130 kDa) and Polox (average molecular weight, Mw ~ 12.6 kDa) polymers were obtained from Merck Ltd. (Budapest, Hungary). The hydroxypropyl-beta-cyclodextrin (degree of substitution ~ 4.5) was the product of Cyclolab Ltd. (Budapest, Hungary). Richter Gedeon Plc kindly supplied the sodium hyaluronate. (Budapest, Hungary). The potassium dihydrogen phosphate, sodium hydroxide, and ethylenediaminetetraacetic acid were purchased from Molar Chemicals Ltd. (Budapest, Hungary). Pharmaceutical-grade distilled water was used to prepare the solution.

### 4.2. Precursor Solution Preparation for the Electrospinning Process

PVA and Polox were chosen as the base of the polymer matrixes. The mass ratio of the polymers was kept constant at 8:2 (m:m). The total polymer concentration (% (*w*/*w*)) of the aqueous solutions was examined at three levels, 10, 12, and 14% (*w*/*w*), and finally it was fixed at 12% (*w*/*w*). PVA solutions were prepared with stirring under heating (80 °C) until clear solutions were achieved. The polox was added to the PVA solutions at room temperature and stirred until complete homogenization. The active ingredient is LEVO, and all precursor solutions contained 3% (*w*/*w*). To increase the solubility of the LEVO, HP-β-CD was used in different molar ratios (drug/CD =1:1, 1:1.5). In some formulations, sodium hyaluronate was also used in two different amounts, 0.2% (*w*/*w*) and 0.4% (*w*/*w*) (Table 7).

### 4.3. Fiber Formation Process

A laboratory-scale electrospinning device (SpinCube, SpinSplit Ltd., Budapest, Hungary) was used to prepare the nanofibrous samples. The precursor solutions were placed in a plastic syringe of 1 mL volume and connected to a 22G needle through a tube. The filled syringe was placed on the pump to provide continuous solution flow. Aluminum foil or baking paper was stuck onto the collector to collect each sample for further analysis. The process was done at ambient conditions of 22 ± 1 °C temperature and 40 ± 5% relative humidity.

### 4.4. Morphological Characterization of the Electrospun Samples

Scanning electron microscopy (SEM) imaging was used to characterize the electrospun samples morphologically. The electrospun samples were fixed by conductive, double-sided carbon adhesive tape and then coated with a thin gold layer (JEOL JFC-1200 Fine Coater, JEOL Ltd., Tokyo, Japan). SEM images were taken with a JEOL JSM-6380LA scanning electron microscope (JEOL Ltd., Tokyo, Japan). The acceleration voltage and the working distance were 10 kV and 10 mm, respectively.

ImageJ v1.46r software (US National Institutes of Health) measured the diameters of 100 individual fibers, and the average with standard deviation was calculated.

### 4.5. Solid-State Characterization of the Samples

#### 4.5.1. FTIR Spectroscopy 

Infrared spectra of the nanofibrous samples and their solid components were recorded using a Jasco FT/IR-4200 spectrophotometer (Jasco Inc., Easton, MD, USA), which was equipped with a Jasco ATR PRO470-H single reflection attenuated total reflectance accessory (ATR). The measurements were performed in absorbance mode, and the FTIR spectra were recorded between 4000 and 400 cm^−1^. One hundred scans were performed at a resolution of 2 cm^−1^ at ambient temperature. The measurements were evaluated using the FTIR software 10.4.3 (Spectra Manager-II, Jasco). As a control for the physicochemical characterization, physical mixtures corresponding to the compositions of the drug-loaded nanofibers (LEVO, Polox, milled PVA, HP-β-CD) were prepared and used.

#### 4.5.2. XRD Measurement

The XRD patterns of the active pharmaceutical ingredient (LEVO), the nanofibrous samples, and the physical mixtures (which were used as a control) were measured on a PANalytical X’Pert3 Powder diffractometer (Malvern Panalytical B.V., The Netherlands) using Cu Kα radiation with a 45 kV accelerating voltage and 40 mA anode current over the range of 4–50 ◦ 2 θ with a 0.0080 ◦ step size and 99.695 s durations per step in reflection mode, spinning the sample holder by 1 s−1. Incident beam optics were as follows: a programmable divergence slit with 15 mm constant irradiated length and an anti-scatter slit at fixed 2◦. Diffracted beam optics consisted of an X’Celerator Scientific ultra-fast line detector with a 0.02 soller slit and a programmable anti-scatter slit with a 15 mm constant observed length. Data were collected by PANalytical Data Collector Application, version 5.5.0.505 (Malvern Panalytical B.V., The Netherlands).

### 4.6. In Vitro Dissolution Study

To mimic the smaller dissolution medium of the eye and consider the parameters of the in-line probe and the linearity obtained from the validation of the UV spectroscopy method for the LEVO, the volume of the dissolution medium (pH 7.4 phosphate buffer) was determined to be 40 mL. The previously measured 8–10 mg fibrous samples were strewed around a stirring bar and put into the sinker of the Jasco dissolution device, which was then placed into a glass bakery (inner diameter: 4 cm). After the probe was immersed, the tempered dissolution fluid was added. The stirring rate was set to 50 rpm. The dissolved drug from the nanofibers was observed for 20 min. The absorbance values were recorded every 10 s. The LEVO concentration was determined spectrophotometrically, based on the calibration curve recorded earlier. The developed method was linear between 1–30 µg/mL LEVO concentration.

### 4.7. In Vivo Cytotoxicity Test

The eye irritation from the developed nanofibers was examined by the hen’s egg test on the chorioallantois membrane. The test is based on observing whether hyperemia, hemorrhage, or coagulation will occur. The developed nanofibrous formulations were measured on the chorioallantois membrane (CAM) of 9-day-old chicken embryos. Fertilized White Leghorn chicken (*Gallus gallus domesticus*) eggs obtained from commercial breeders (Prophyl-BIOVO Hungary Ltd., Mohacs) were used for the test. The eggs were maintained at a temperature of 37.5 ± 0.5 °C in a humidified HEKA 1+ egg incubator (Rietberg, Germany). To prepare the chick embryo for the in vivo cytotoxicity test, as a first step, the hard shell of the egg was removed in a 1 cm^2^ area using ophthalmic surgical scissors. Next, the inner soft membrane was removed to expose the highly vascularized CAM. The controls and LEVO-loaded nanofiber sheets were placed on the surface of the vascularized CAM. Phosphate-buffered saline (PBS) pH 7.4 was used as a negative control, while 0.1N NaOH solution was applied as a positive control. To evaluate the irritation effects, images were captured at 0.5, 2, and 5 min using a Nikon SMZ25 stereomicroscope (Unicam Ltd., Budapest, Hungary). Image processing was conducted using Nikon’s proprietary software, QCapture Pro 2 (NIS-Elements Basic Research version obtained from AURO-SCIENCE Consulting Ltd., Budapest, Hungary).

Based on the article of Luepke et al., a scoring system was used to quantify the irritation response observed on the chorioallantoic membrane (CAM) [36]. The most used scoring method is the irritation score, based on the time it takes for specific reactions to occur after applying the test substance: hyperaemia, haemorrhage, and coagulation. The CAM, the blood vessels, the capillary system, and the albumen were examined and scored for irritant effects at 0.5, 2, and 5 min after treatment. The numerical time-dependent scores for hyperaemia, haemorrhage, and coagulation (Table 8) are summed to give a single numerical value indicating the irritation potential of the test substance on a scale with a maximum value of 21. Table 9 summarizes the cumulative scores and the belonging irritation assessments.

### 4.8. Aseptic Sample Preparations

The ocular application of the nanofibrous insert requires a sterile product. To reach this purpose, the precursor solutions were filtered through a polyether sulfone-based, sterile membrane filter of 0.21 µm pore size, which was sterilized by gamma irradiation (Sarsted Ltd.). The spinning chamber, the fittings, the needle, the syringe, and the tube were prewashed with ethanol (70%, Molar Chemicals Ltd.). The electrospinning device was equipped with a UV-Lampe, which worked for 20 min. After that, samples were taken with sterile sampling sticks from eight different places in the chamber for further microbiological analysis. The test was carried out based on the requirements of Hungarian Pharmacopeia. The applicability of the membrane filter was tested by UV spectroscopy. The spectra of unfiltered and filtered precursor solutions were recorded between 200–800 nm to test whether the filter might bind the drug.

### 4.9. Antibacterial Study 

The sensitivity of ATCC strains of bacteria causing common eye infections to the LEVO, LEVO-cyclodextrin solutions, commercially available LEVO-containing eye drops, and four different fibrous formulations were investigated.

Using the disk diffusion method, the sensitivity of clinical isolates (*S. pneumoniae*, *H. parainfluenzae*, *M. catarrhalis*, and two *N. gonorrhoeae*) was compared between four different types of disks and solutions containing the same active ingredients as the disks. The disks were 6 mm in diameter, the same diameter achieved by dropping 5 µLof the solution onto an agar plate. These solution volumes contained the amounts of active ingredients listed in the table. The bacterial lawns were prepared from 0.5 McFarland turbidity suspensions on chocolate + polyvitex agar, and the results were read after 24 h of incubation.

The efficacy of the prepared compounds was determined using the broth microdilution method. Bacterial strains (*E. coli* ATCC 25218, *P. aeruginosa* ATCC 27853, and *S. aureus* ATCC 29213) were grown on Mueller-Hinton MH agar plates at 35 °C overnight. Appropriate numbers of colonies were suspended in physiological saline to reach the density of 0.5 McFarland for inoculation. The given preparations of LEVO were two-fold and were serially diluted from 256 to 0.5 μg/mL in MH broth, and then 100 μL of each dilution was transferred into microplate holes. Inoculation was carried out with 10 μL of each bacterial suspension. Incubation was performed at 37 °C for 24 h, and the determination of the minimal inhibitory concentration (MIC) was made with the naked eye.

## 5. Conclusions

The study demonstrated that the formation of clear, fibrous structures containing LEVO was enabled by using different concentrations of HP-β-CD and sodium hyaluronate in PVA-Polox viscous precursor solutions. Physicochemical analyses (FTIR and XRD) confirmed the presence of LEVO in an amorphous state within the fibers, a characteristic that is essential for achieving rapid drug release. This fast release is particularly advantageous for ophthalmic applications, as it ensures a high initial concentration of the drug at the site of infection. Even at higher levels, HP-β-CD did not significantly hinder the fast drug release.

The nanofibrous formulations, especially the LEVO complex, enhanced antimicrobial activity, with the efficacy varying depending on the bacterial strain. This suggests that these formulations can be tailored for targeted antimicrobial therapy. The F7 nanofibrous formulation was identified as particularly promising due to its rapid and complete dissolution, good biocompatibility, and effective antimicrobial properties. Its gelling properties indicate that the residence time on the eye’s surface can be increased, potentially reducing discomfort and enhancing therapeutic outcomes.

Overall, the potential of LEVO-loaded nanofibers as an alternative to conventional eye drops was demonstrated. The nanofibers matched the antimicrobial efficacy of eye drops while offering additional advantages, such as enhanced bioavailability, a preservative-free composition, and the potential for improved patient adherence. The findings underscore the importance of balancing efficacy and safety, achieved through carefully optimizing excipients like HP-β-CD and sodium hyaluronate. Further in vivo studies are recommended to verify these formulations’ long-term safety and therapeutic efficacy, which could lead to their clinical application in ocular drug delivery.

## Figures and Tables

**Table 1:** The optimized electrospinning process parameters for the fiber formation of the different precursor solutions (F1–F9).

Formulation Code	Flow Rate (µL/s)	Voltage (kV)	Effective Distance (cm)
F1	0.08	16.8	12.5
F2	0.08	17.2	12.5
F3	0.08	18.8	12.5
F4	0.1	18.9	12.5
F5	0.1	19	12.5
F6	0.15	22.3	12.5
F7	0.15	23.5	12.5
F8	0.15	22.7	12.5
F9	0.15	23.5	12.5

**Table 2:** The average fiber diameters with standard deviations (SD) and the morphological evaluations of the prepared electrospun samples of different compositions.

Formulation Code	Average Fiber Diameter ± SD (nm)	Morphological Characteristics
F1	123 ± 35	fibrous, but with beads
F2	149 ± 33	fibrous
F3	183 ± 31	fibrous
F4	239 ± 40	fibrous
F5	304 ± 59	fibrous
F6	237 ± 36	fibrous
F7	286 ± 42	fibrous
F8	164 ± 31	fibrous
F9	249 ± 43	fibrous

**Table 3:** Cumulative scores and assessments of irritation potential of different levofloxacin-loaded nanofibrous samples tested in the chorioallantoic membrane test.

Sample Name	Cumulative Score	Irritation Assessment
F6_NF	0	practically none
F7_NF	7	moderate
F8_NF	0	practically none
F9_NF	7	moderate

**Table 4:** The diameter of the inhibition zones in the case of different bacterial strains (*Streptococcus pneumoniae* (*S. pneumoniae*), *Moraxella catarrhalis* (*M. catarrhalis*), *Haemophilus parainfluenzae* (*H. parainfluenzae*), and two strains of *Neisseria gonorrhoeae* (*N. gonorrhoeae*).

	Diameter (mm)						
	LEVO	LEVO:CD 1:1 (n:n)	LEVO:CD 1:1.5 (n:n)	F6_NF	F8_NF	F7_NF	F9_NF
*S. pneumoniae*	30	32	30	32	34	36	34
*M. catarrhalis*	38	40	40	40	46	44	42
*H. parainfluenzae*	30	30	28	32	32	30	32
*N. gonorrhoeae 1*	38	40	40	40	40	40	38
*N. gonorrhoeae 2*	56	56	56	56	56	56	56

**Table 5:** Minimal Inhibitory Concentration (MIC) values of various levofloxacin (LEVO)-containing solutions (LEVO, LEVO:CD 1:1 (n:n), and LEVO:CD 1:1.5 (n:n)), the commercially available eye drop, and the nanofibrous (NF) formulations (F6–F9) (where LEVO: levofloxacin, CD: hydroxypropyl-beta-cyclodextrin, −: negative, and +: positive, *E. coli*: *Escherichia coli*, *S. aureus: Staphylococcus aureus,* and *P. aeruginosa: Pseudomonas aeruginosa*).

**MIC (µg/mL)**	**LEVO**												
		128	64	32	16	8	4	2	1	0.5	0.25	NC	PC
<0.25	*E. coli*	-	-	-	-	-	-	-	-	-	-	✓	✓
<0.25	*E. coli*	-	-	-	-	-	-	-	-	-	-	✓	✓
16	*S. aureus*	-	-	-	-	+	+	+	+	+	+	✓	✓
8	*S. aureus*	-	-	-	-	-	+	+	+	+	+	✓	✓
4	*P. aeruginosa*	-	-	-	-	-	-	+	+	+	+	✓	✓
4	*P. aeruginosa*	-	-	-	-	-	-	+	+	+	+	✓	✓
**MIC (µg/mL)**	**Solution of LEVO:CD 1:1 (n:n)**												
		32	16	8	4	2	1	0.5	0.25	0.125	0.0625	NC	PC
<0.0625	*E. coli*	-	-	-	-	-	-	-	-	-	-	✓	✓
<0.0625	*E. coli*	-	-	-	-	-	-	-	-	-	-	✓	✓
0.5	*S. aureus*	-	-	-	-	-	-	-	+	+	+	✓	✓
1	*S. aureus*	-	-	-	-	-	-	+	+	+	+	✓	✓
4	*P. aeruginosa*	-	-	-	-	+	+	+	+	+	+	✓	✓
4	*P. aeruginosa*	-	-	-	-	+	+	+	+	+	+	✓	✓
**MIC (µg/mL)**	**Solution of LEVO:CD 1:1.5 (n:n)**												
		32	16	8	4	2	1	0.5	0.25	0.125	0.0625	NC	PC
<0.0625	*E. coli*	-	-	-	-	-	-	-	-	-	-	✓	✓
<0.0625	*E. coli*	-	-	-	-	-	-	-	-	-	-	✓	✓
1	*S. aureus*	-	-	-	-	-	-	+	+	+	+	✓	✓
1	*S. aureus*	-	-	-	-	-	-	+	+	+	+	✓	✓
8	*P. aeruginosa*	-	-	-	+	+	+	+	+	+	+	✓	✓
8	*P. aeruginosa*	-	-	-	+	+	+	+	+	+	+	✓	✓
**MIC (µg/mL)**	**Eye drop**												
		64	32	16	8	4	2	1	0.5	0.25	0.125	NC	PC
<0.125	*E. coli*	-	-	-	-	-	-	-	-	-	-	✓	✓
<0.125	*E. coli*	-	-	-	-	-	-	-	-	-	-	✓	✓
0.25	*S. aureus*	-	-	-	-	-	-	-	-	-	+	✓	✓
0.25	*S. aureus*	-	-	-	-	-	-	-	-	-	+	✓	✓
1	*P. aeruginosa*	-	-	-	-	-	-	-	+	+	+	✓	✓
1	*P. aeruginosa*	-	-	-	-	-	-	-	+	+	+	✓	✓
**MIC (µg/mL)**	**F6 nanofibrous sample**												
		64	32	16	8	4	2	1	0.5	0.25	0.125	NC	PC
<0.125	*E. coli*	-	-	-	-	-	-	-	-	-	-	✓	✓
<0.125	*E. coli*	-	-	-	-	-	-	-	-	-	-	✓	✓
16	*S. aureus*	-	-	-	+	+	+	+	+	+	+	CONT	✓
16	*S. aureus*	-	-	-	+	+	+	+	+	+	+	✓	✓
4	*P. aeruginosa*	-	-	-	-	-	+	+	+	+	+	✓	✓
4	*P. aeruginosa*	-	-	-	-	-	+	+	+	+	+	✓	✓
**MIC (µg/mL)**	**F8 nanofibrous sample**												
		64	32	16	8	4	2	1	0.5	0.25	0.125	NC	PC
<0.125	*E. coli*	-	-	-	-	-	-	-	-	-	-	✓	✓
<0.125	*E. coli*	-	-	-	-	-	-	-	-	-	-	✓	✓
0.25	*S. aureus*	-	-	-	-	-	-	-	-	-	+	✓	✓
0.25	*S. aureus*	-	-	-	-	-	-	-	-	-	+	CONT	✓
4	*P. aeruginosa*	-	-	-	-	-	+	+	+	+	+	✓	✓
4	*P. aeruginosa*	-	-	-	-	-	+	+	+	+	+	✓	✓
**MIC (µg/mL)**	**F7 nanofibrous sample**												
		64	32	16	8	4	2	1	0.5	0.25	0.125	NC	PC
<0.125	*E. coli*	-	-	-	-	-	-	-	-	-	-	✓	✓
<0.125	*E. coli*	-	-	-	-	-	-	-	-	-	-	✓	✓
0.5	*S. aureus*	-	-	-	-	-	-	-	-	+	+	✓	✓
0.5	*S. aureus*	-	-	-	-	-	-	-	-	+	+	✓	✓
4	*P. aeruginosa*	-	-	-	-	-	+	+	+	+	+	✓	✓
4	*P. aeruginosa*	-	-	-	-	-	+	+	+	+	+	✓	✓
**MIC (µg/mL)**	**F9 nanofibrous sample**												
		64	32	16	8	4	2	1	0.5	0.25	0.125	NC	PC
<0.125	*E. coli*	-	-	-	-	-	-	-	-	-	-	✓	✓
<0.125	*E. coli*	-	-	-	-	-	-	-	-	-	-	✓	✓
0.5	*S. aureus*	-	-	-	-	-	-	-	-	+	+	✓	✓
0.5	*S. aureus*	-	-	-	-	-	-	-	-	+	+	✓	✓
4	*P. aeruginosa*	-	-	-	-	-	+	+	+	+	+	✓	✓
4	*P. aeruginosa*	-	-	-	-	-	+	+	+	+	+	✓	✓

**Table 6:** The minimum bactericidal concentration (MBC) of various levofloxacin (LEVO)-containing solutions (LEVO, LEVO:CD 1:1 (n:n), and LEVO:CD 1:1.5 (n:n)), the commercially available eye drop, and the nanofibrous formulations (F6-F9) (where *E. coli*: *Escherichia coli, S. aureus: Staphylococcus aureus,* and *P. aeruginosa: Pseudomonas aeruginosa*).

MBC (µg/mL)								
	LEVO	Solution of LEVO:CD 1:1 (n:n)	Solution of LEVO:CD 1:1.5 (n:n)	Eye Drop	F6	F8	F7	F9
*E. coli*	4	8	8	4	8	8	4	8
*S. aureus*	32	32	32	32	32	64	64	64
*P. aeruginosa*	8	8	8	1	8	8	8	8

**Table 7:** Composition of the different aqueous precursor solutions used for the electrospinning process (The abbreviations are the following: PVA: polyvinyl alcohol, Polox: Poloxamer 407, LEVO: levofloxacin, and HP-β-CD: hydroxypropyl-beta-cyclodextrin).

Formulation Code	PVA:Polox (m:m)	Total Polymer Concentration (% (*w*/*w*))	LEVO (% (*w*/*w*))	LEVO: HP-β-CD (n:n)	Sodium Hyaluronate (% (*w*/*w*))
F1	8:2	10	0	0	0
F2	8:2	12	0	0	0
F3	8:2	14	0	0	0
F4	8:2	12	3	1:1	0
F5	8:2	12	3	1:1.5	0
F6	8:2	12	3	1:1	0.2
F7	8:2	12	3	1:1.5	0.2
F8	8:2	12	3	1:1	0.4
F9	8:2	12	3	1:1.5	0.4

**Table 8:** Scoring scheme for irritation testing with the hen’s egg chorioallantoic membrane.

Effect		Score		
	Time (min)	0.5	2	5
hyperaemia		5	3	1
haemorrhage		7	5	3
coagulation		9	7	5

**Table 9:** Classification of cumulative scores in the chorioallantoic membrane test.

Cummulative Score	Irritation Assessment
0–0.9	practically none
1–4.9	slight
5–8.9	moderate
9–21	strong

## Data Availability

Data reported in the study are available in the manuscript.

## List of Abbreviations

Attenuated total reflectance accessory (ATR), chorioallantois membrane (CAM), cyclodextrin (CD), *Escherichia coli (E. coli),* Fourier Transform Infrared (FTIR), *Haemophilus parainfluenzae (H. parainfluenzae),* hydroxypropyl-beta-cyclodextrin (HP-β-CD), levofloxacin (LEVO), minimal inhibitory concentration (MIC), Minimum Bactericidal Concentration (MBC), *Moraxella catarrhalis (M. catarrhalis),* Mueller-Hinton (MH), *Neisseria gonorrhoeae (N. gonorrhoeae),* Poloxamer 407 (Polox), polyvinyl alcohol (PVA), *Pseudomonas aeruginosa (P. aeruginosa),* scanning electron microscopy (SEM), *Staphylococcus aureus (S. aureus), Streptococcus pneumoniae (S. pneumoniae),* sulfobutyl-ether-beta-cyclodextrin (SBE-β-CD), X-ray diffraction (XRD).

## References

[1] Palioura S., Gibbons A., Miller D., O’Brien T.P., Alfonso E.C., Spierer O. (2018). Clinical Features, Antibiotic Susceptibility Profile, and Outcomes of Infectious Keratitis Caused by *Stenotrophomonas maltophilia*. Cornea.

[2] Sharma A., Taniguchi J. (2017). Review: Emerging strategies for antimicrobial drug delivery to the ocular surface: Implications for infectious keratitis. Ocul. Surf..

[3] Oum B.S., Kim N.M., Lee J.S., Park Y.M. (2014). Effects of Fluoroquinolone Eye Solutions without Preservatives on Human Corneal Epithelial Cells in vitro. Ophthalmic Res..

[4] Bezwada P., Clark L.A., Schneider S. (2008). Intrinsic cytotoxic effects of fluoroquinolones on human corneal keratocytes and endothelial cells. Curr. Med. Res. Opin..

[5] Keating G.M. (2009). Levofloxacin 0.5% Ophthalmic Solution. Drugs.

[6] Li G., Xu L., Jiang M., Wu X. (2020). Eye drops and eye gels of levofloxacin: Comparison of ocular absorption characterizations and therapeutic effects in the treatment of bacterial keratitis in rabbits. Drug Dev. Ind. Pharm..

[7] An J.A., Kasner O., Samek D.A., Lévesque V. (2014). Evaluation of eyedrop administration by inexperienced patients after cataract surgery. J. Cataract Refract. Surg..

[8] Hermann M.M., Üstündag C., Diestelhorst M. (2010). Electronic compliance monitoring of topical treatment after ophthalmic surgery. Int. Ophthalmol..

[9] Bamiro O.A., Ubale R.V., Addo R.T., Addo R.T. (2016). Background of Ocular Drug Delivery. Ocular Drug Delivery: Advances, Challenges and Applications.

[10] Lanier O.L., Manfre M.G., Bailey C., Liu Z., Sparks Z., Kulkarni S., Chauhan A. (2021). Review of Approaches for Increasing Ophthalmic Bioavailability for Eye Drop Formulations. AAPS PharmSciTech.

[11] Kassem M.A., Abdel Rahman A.A., Ghorab M.M., Ahmed M.B., Khalil R.M. (2007). Nanosuspension as an ophthalmic delivery system for certain glucocorticoid drugs. Int. J. Pharm..

[12] Alvarez-Lorenzo C., Anguiano-Igea S., Varela-García A., Vivero-Lopez M., Concheiro A. (2019). Bioinspired hydrogels for drug-eluting contact lenses. Acta Biomater..

[13] Gaballa S.A., Kompella U.B., Elgarhy O., Alqahtani A.M., Pierscionek B., Alany R.G., Abdelkader H. (2021). Corticosteroids in ophthalmology: Drug delivery innovations, pharmacology, clinical applications, and future perspectives. Drug Deliv. Transl. Res..

[14] Kim H.M., Woo S.J. (2021). Ocular Drug Delivery to the Retina: Current Innovations and Future Perspectives. Pharmaceutics.

[15] Maulvi F.A., Desai D.T., Shetty K.H., Shah D.O., Willcox M.D.P. (2021). Advances and challenges in the nanoparticles-laden contact lenses for ocular drug delivery. Int. J. Pharm..

[16] Terreni E., Burgalassi S., Chetoni P., Tampucci S., Zucchetti E., Fais R., Ghelardi E., Lupetti A., Monti D. (2020). Development and Characterization of a Novel Peptide-Loaded Antimicrobial Ocular Insert. Biomolecules.

[17] Ghezzi M., Ferraboschi I., Fantini A., Pescina S., Padula C., Santi P., Sissa C., Nicoli S. (2023). Hyaluronic acid–PVA films for the simultaneous delivery of dexamethasone and levofloxacin to ocular tissues. Int. J. Pharm..

[18] Gupta H., Aqil M., Khar R.K., Ali A., Bhatnagar A., Mittal G. (2011). Biodegradable levofloxacin nanoparticles for sustained ocular drug delivery. J. Drug Target..

[19] Chang Y.-F., Cheng Y.-H., Ko Y.-C., Chiou S.-H., Jui-Ling Liu C. (2022). Development of topical chitosan/β-glycerophosphate-based hydrogel loaded with levofloxacin in the treatment of keratitis: An ex-vivo study. Heliyon.

[20] Ameeduzzafar, Imam S.S., Abbas Bukhari S.N., Ahmad J., Ali A. (2018). Formulation and optimization of levofloxacin loaded chitosan nanoparticle for ocular delivery: In-vitro characterization, ocular tolerance and antibacterial activity. Int. J. Biol. Macromol..

[21] Abd El-Bary A., Kamal Ibrahim H., Haza’a B.S., Al Sharabi I. (2019). Formulation of sustained release bioadhesive minitablets containing solid dispersion of levofloxacin for once daily ocular use. Pharm. Dev. Technol..

[22] Ameeduzzafar, Khan N., Alruwaili N.K., Bukhari S.N.A., Alsuwayt B., Afzal M., Akhter S., Yasir M., Elmowafy M., Shalaby K. (2020). Improvement of Ocular Efficacy of Levofloxacin by Bioadhesive Chitosan Coated PLGA Nanoparticles: Box-behnken Design, In-vitro Characterization, Antibacterial Evaluation and Scintigraphy Study. Iran. J. Pharm. Res..

[23] De Gaetano F., Marino A., Marchetta A., Bongiorno C., Zagami R., Cristiano M.C., Paolino D., Pistarà V., Ventura C.A. (2021). Development of Chitosan/Cyclodextrin Nanospheres for Levofloxacin Ocular Delivery. Pharmaceutics.

[24] Jain P., Jaiswal C.P., Mirza M.A., Anwer M.K., Iqbal Z. (2020). Preparation of levofloxacin loaded in situ gel for sustained ocular delivery: In vitro and ex vivo evaluations. Drug Dev. Ind. Pharm..

[25] Shi H., Wang Y., Bao Z., Lin D., Liu H., Yu A., Lei L., Li X., Xu X. (2019). Thermosensitive glycol chitosan-based hydrogel as a topical ocular drug delivery system for enhanced ocular bioavailability. Int. J. Pharm..

[26] Thakkar R., Komanduri N., Dudhipala N., Tripathi S., Repka M.A., Majumdar S. (2021). Development and optimization of hot-melt extruded moxifloxacin hydrochloride inserts, for ocular applications, using the design of experiments. Int. J. Pharm..

[27] Polat H.K., Bozdağ Pehlivan S., Özkul C., Çalamak S., Öztürk N., Aytekin E., Fırat A., Ulubayram K., Kocabeyoğlu S., İrkeç M. (2020). Development of besifloxacin HCl loaded nanofibrous ocular inserts for the treatment of bacterial keratitis: In vitro, ex vivo and in vivo evaluation. Int. J. Pharm..

[28] Zhang Y.Z., Venugopal J., Huang Z.M., Lim C.T., Ramakrishna S. (2006). Crosslinking of the electrospun gelatin nanofibers. Polymer.

[29] Singla J., Bajaj T., Goyal A.K., Rath G. (2019). Development of Nanofibrous Ocular Insert for Retinal Delivery of Fluocinolone Acetonide. Curr. Eye Res..

[30] Cui W., Zhou Y., Chang J. (2010). Electrospun nanofibrous materials for tissue engineering and drug delivery. Sci. Technol. Adv. Mater..

[31] Fan J., Li G., Deng S., Wang Z. (2019). Mechanical Properties and Microstructure of Polyvinyl Alcohol (PVA) Modified Cement Mortar. Appl. Sci..

[32] Almeida H., Amaral M.H., Lobão P., Sousa Lobo J.M. (2013). Applications of poloxamers in ophthalmic pharmaceutical formulations: An overview. Expert Opin. Drug Deliv..

[33] Miranda G.M., Santos V.O., Bessa J.R., Teles Y.C.F., Yahouédéhou S.C., Goncalves M.S., Ribeiro-Filho J. (2021). Inclusion Complexes of Non-Steroidal Anti-Inflammatory Drugs with Cyclodextrins: A Systematic Review. Biomolecules.

[34] Grassiri B., Knoll P., Fabiano A., Piras A.M., Zambito Y., Bernkop-Schnürch A. (2022). Thiolated Hydroxypropyl-β-cyclodextrin: A Potential Multifunctional Excipient for Ocular Drug Delivery. Int. J. Mol. Sci..

[35] He Z.-X., Wang Z.-H., Zhang H.-H., Pan X., Su W.-R., Liang D., Wu C.-B. (2011). Doxycycline and hydroxypropyl-β-cyclodextrin complex in poloxamer thermal sensitive hydrogel for ophthalmic delivery. Acta Pharm. Sin. B.

[36] Luepke N.P. (1985). Hen’s egg chorioallantoic membrane test for irritation potential. Food Chem. Toxicol..

